# Ultraviolet-B induces ERCC6 repression in lens epithelium cells of age-related nuclear cataract through coordinated DNA hypermethylation and histone deacetylation

**DOI:** 10.1186/s13148-016-0229-y

**Published:** 2016-05-26

**Authors:** Yong Wang, Fei Li, Guowei Zhang, Lihua Kang, Huaijin Guan

**Affiliations:** Eye Institute, Affiliated Hospital of Nantong University, 20 Xisi Road, Nantong, Jiangsu China; Ophthalmology Department, Chengdu Fifth People’s Hospital, Chengdu, Sichuan China

**Keywords:** *ERCC6*, Age-related nuclear cataract (ARNC), Lens epithelial cells (LECs), DNA methylation, Histone deacetylation, Ultraviolet (UV), Sp1

## Abstract

**Background:**

Ultraviolet-B (UVB) exposure attributes to the formation of age-related nuclear cataract (ARNC), which is mediated with DNA damage. DNA damage, an important factor for pathogenesis of ARNC, is induced by UVB, and is generally resolved by the nucleotide excision repair (NER) repair mechanism. Cockayne syndrome complementation group B (CSB) protein coded by *ERCC6* is a vital component for NER. However, we found no association between selected *ERCC6 *polymorphisms and ARNC. In this study, we investigated whether UVB exposure could alter *ERCC6* expression and the process could involve epigenetic changes of DNA methylation and/or histone acetylation of *ERCC6* in the lens epithelial cells (LECs). We also assessed the involvement of those coordinated changes in lens tissue from ARNC patients.

**Results:**

mRNA and protein expression of *ERCC6* in lens tissue (LECs) were lower in ARNCs than those in the controls. This reduction corresponded to methylation of a CpG site at the *ERCC6* promoter and histone modifications (methylation and acetylation) nearby this site. UVB-treated human lens epithelium B3 (HLE-B3) and 239T cell presented (1) increased apoptosis, suggesting reduced UV-damage repair, (2) hypermethylation of the CpG site located at position -441 (relative to transcription start site) within the binding region for transcriptional factor Sp1 in the *ERCC6* promoter, (3) the enhancement of histone H3K9 deacetylation, (4) induction in DNA methyltransferases 3b (DNMT3b) and histone deacetylase1 (HDAC1) associated to the CpG site of *ERCC6* by CHIP assay.

**Conclusions:**

These findings suggest an orchestrated mechanism triggered by UVB radiation where the concurrent association of specific hypermethylation CpG site, H3K9 deacetylation of *ERCC6*, and repression of *ERCC6* gene expression. Taken together, with the similar changes in the lens tissue from ARNC patients, our data unveiled a possible mechanism of epigenetic modification of DNA repair gene in the pathogenesis of ARNC.

**Electronic supplementary material:**

The online version of this article (doi:10.1186/s13148-016-0229-y) contains supplementary material, which is available to authorized users.

## Background

Age-related cataract (ARC) is a leading cause of visual impairment worldwide [1–5]. Age-related nuclear cataract (ARNC) ranks as the most common type of ARC [5]. Several studies suggested that ultraviolet (UV) radiation may be a cataractogenic factor [6–10]. UV radiation that reaches the surface of earth consists of two components: UVA (315–400 nm) and ultraviolet-B (UVB) (280–315 nm) [11]. UVB is particularly relevant to the formation and development of cataract, since the energy of UVB is substantially absorbed to injure the lens [12]. The wavelength range around 300 nm of UVB is most harmful for the lens [13]. Several studies showed a correlation between UVB exposure and nuclear cataract formation [14–16]. However, the exact mechanism is not completely understood in such cataractogenesis.

Exposure of the lens to UVB induces DNA lesion and oxidative stress [11, 17]. Oxidative stress-induced DNA damage is considered an important factor in the pathogenesis of ARNCs [18, 19]. There are several pathways involved in DNA repair, including nucleotide excision repair (NER) and base excision repair (BER) [20]. NER is a vital excision mechanism that removes UV-induce DNA damage [21, 22]. In the NER process, Cockayne syndrome complementation group B (CSB) protein (coded by *ERCC6*) recruits NER repair factors to the DNA damage site and removes DNA lesions. Defects in the CSB protein were found to be involved in the Cockayne syndrome (CS) [23]. Cockayne syndrome patients with CSB mutations were found to suffer from severe cataract [24]. But, our previous study did not find the association between selected *ERCC6* polymorphisms (rs4838519 and rs4253038) and ARNCs [25]. Recently, our study showed that hypermethylation of *OGG1* gene links to low expression of *OGG1* and ARCs formation [26]. *OGG1* plays a vital role in the BER pathway of DNA repair [26]. This prompted us to study the possible epigenetic mechanisms for the regulation of *ERCC6* expression in lens epithelium cells (LECs) of ARNCs.

Alterations in DNA methylation status and chromatin structure by histone modification represent the major epigenetic mechanisms implicated in the regulation of gene transcription without alteration of the DNA sequence [27, 28]. Many human genes contain CpG-rich regions (CpG islands) near their transcription start sites and are normally unmethylated. Methylation of cytosine of a CpG dinucleotide is catalyzed and maintained by DNA methyltransferases DNMTs (DNMT1, DNMT3a, and DNMT3b) and results in repression of gene expression [29]. Histone deacetylation is catalyzed by histone deacetylase (HDACs) including ClassIHDAC, ClassIIHDAC, ClassIII HDAC, and Class IV HDAC. Class I HDACs include HDAC1, HDAC2, HDAC3, and HDAC8 [30]. Evidences suggest that the DNA methylation and histone modification are strictly linked and can reciprocally associate or interfere [31, 32]. A study showed that DNMT3b can act as transcriptional repressors by using their ATRX domain to recruit HDAC1 [33]. Several researches also showed that the binding of the transcription factor at the promoter of several genes are regulated by histone acetylation and DNA methylation [34–36]. Methylation status of the transcription factor Sp1 binding site at KCNMB1 promoter adjusts the gene expression, which is a novel mechanism of DNA demethylation in a sequence-specific manner at transcription factor-binding elements in the gene promoter region [37].

UVB can also induce altered methylation of genes [38, 39], but there is still little understanding in specific CpG site methylation crucial for the repression of the gene expression. Here, we aimed at evaluating whether epigenetic events at a special site play a crucial role in the UVB-induced transcriptional inactivation of *ERCC6* in an in intro model and LECs of ARNCs. We investigated the functional relevance of DNA methylation status and histone modifications in the regulation of *ERCC6* gene expression. We found that UVB-treated human lens epithelium B3 (HLE-B3) and 239T cell caused an orchestrated epigenetic and transcriptomic changes in the context of ERCC6 gene. Similar changes were also observed in human lens tissue of ARNCs collected from cataract surgery.

## Results

### mRNA and protein expression of *ERCC6* in lens tissue (LECs) of controls and ARNCs

**Fig. 1:**
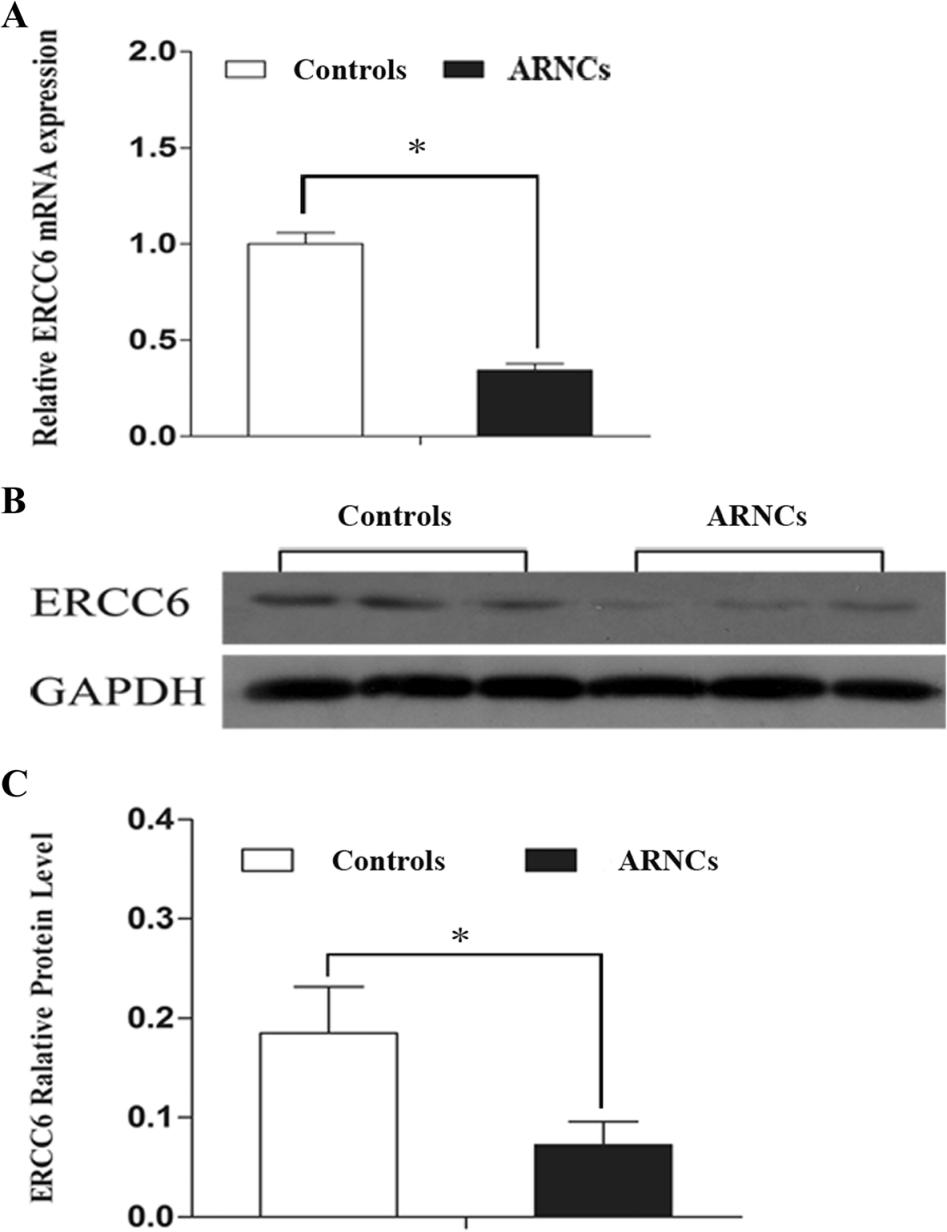
Relative expression of mRNA and protein levels of *ERCC6* in LECs of controls and ARNCs. **a** qRT-PCR analysis of the mRNA expression of *ERCC6* in LECs of controls and ARNCs. The mRNA levels were normalized by using the GAPDH as the inner control. Data were depicted as the mean ± SD of three independent experiments. *P < 0.01. **b** The amount of ERCC6 protein in LECs of controls and ARNCs was measured by Western bolt analysis (Samples labeled as #1–#15 of the controls and #1–#15 of the ARNCs out of total 30 samples in each group). **c** Relative *ERCC6* protein level to GAPDH is presented as mean ± SD. *P < 0.01

The transcript and protein expression of *ERCC6* in LECs of controls and ARNCs were detected by quantitative reverse-transcription polymerase chain reaction (qRT-PCR) and Western blot analysis. mRNA expression of *ERCC6* was 2.44-fold lower in LECs of ARNC than that of the controls (Fig. 1a, Additional file 1: Table S1). Lower protein levels of *ERCC6* were also detected in LECs of ARNCs than the controls (Fig. 1b, c).

### *ERCC6* mRNA levels and protein expression correlate with the methylation status of CpG site 8 in LECs of controls and ARNCs

**Fig. 2:**
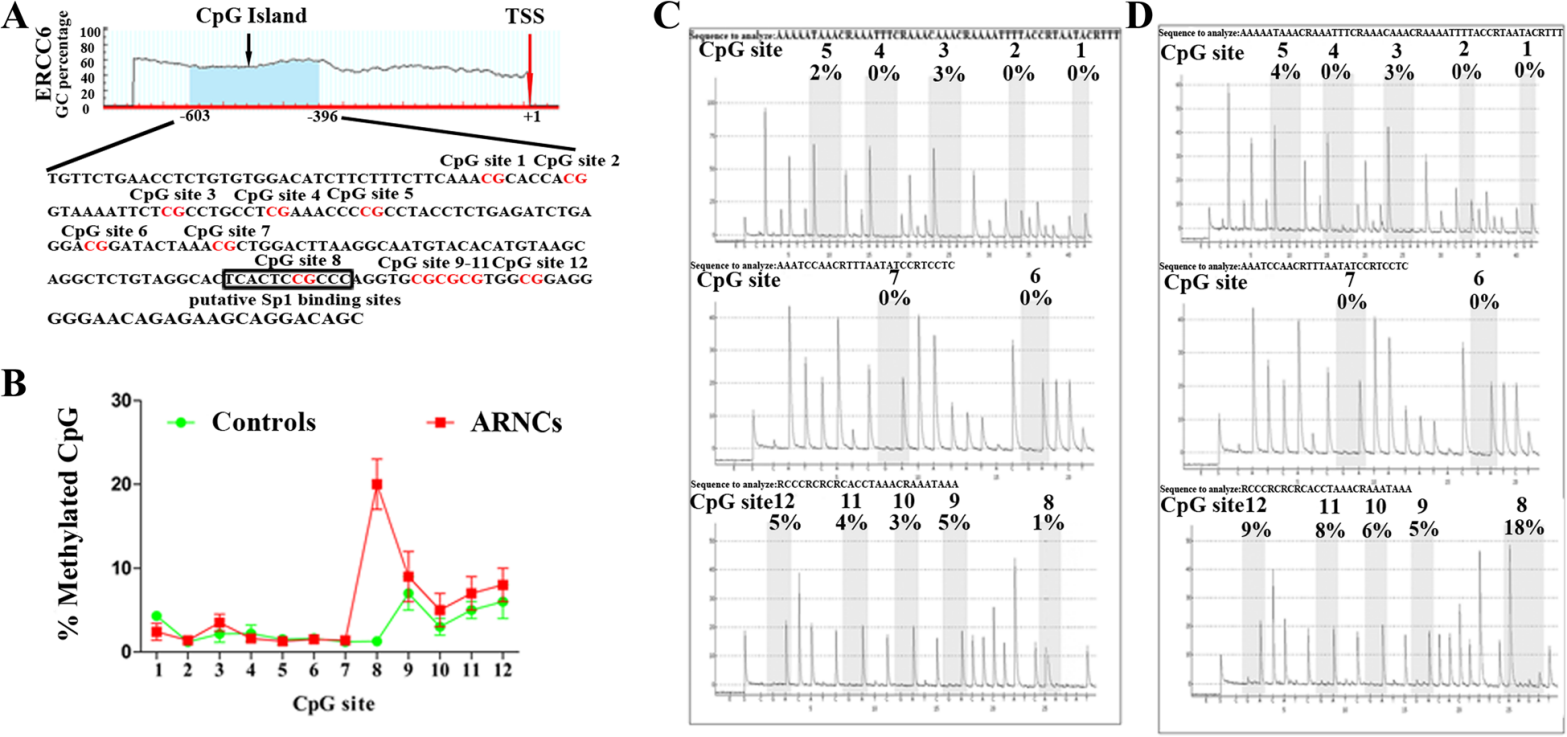
Methylation status of the CpG island at promoter of *ERCC6* in LECs of controls and ARNCs (30 controls and 30 ARNCs, the samples cover those analyzed in Fig. 1). **a** The CpG island (-603/-396, *black arrow*) located in the promoter of *ERCC6*. There are twelve CpG sites (*the red marker letter*) and a putative binding site for Sp1 (-446/-437) relative to transcriptional starting site (*red arrow*) in the region. **b** In LECs of ARNCs, the CpG site 8 displayed hypermethylation compared to the controls. *P < 0.01. **c** Representative pyrosequencing results of the ARNC. **d** Representative pyrosequencing results of the control

Bioinformatic analysis indicated a CpG island (from -603 to -396, transcription start site (TSS) as +1: chr10:49540952) located in the promoter of *ERCC6*, and a putative binding site for Sp1 (from -446 to -437) was predicted in this region (Fig. 2a). We examined 60 specimens (30 controls and 30 ARNCs, the samples cover those analyzed in Additional file 2: Figure S1. Additional file 1: Table S1.) to determine the methylation pattern of the *ERCC6* promoter. Twelve CpG sites located at -564 (site 1), -557 (site 2), -545 (site 3), -536 (site 4), -528 (site 5), -505 (site 6), -494 (site 7), -441 (site 8), -431 (site 9), -429 (site 10), -427 (site 11), and -422 (site 12) from the TSS (Fig. 2a) were analyzed by pyrosequencing. As shown in Fig. 2b, methylation rates in those 12 sites were 2.4, 1.4, 3.5, 1.6, 1.3, 1.5, 1.4, 20.6, 10.9, 4.9, 6.4, and 7.6 %, respectively, in ARNC group and 4.3, 1.2, 2.17, 2.2, 1.5, 1.6, 1.2, 1.3, 7.5, 3.5, 4.2, and 5.6 % in the controls. Representative pyrosequencing results of a control (Fig. 2b) and an ARNC were showed in Fig. 2c.

Further analysis showed *ERCC6* mRNA and protein expression levels were correlated with the methylation status of CpG site 8 in LECs of controls and ARNCs (*P* < 0.01).

### Identification of *ERCC6* minimal promoter

**Fig. 3:**
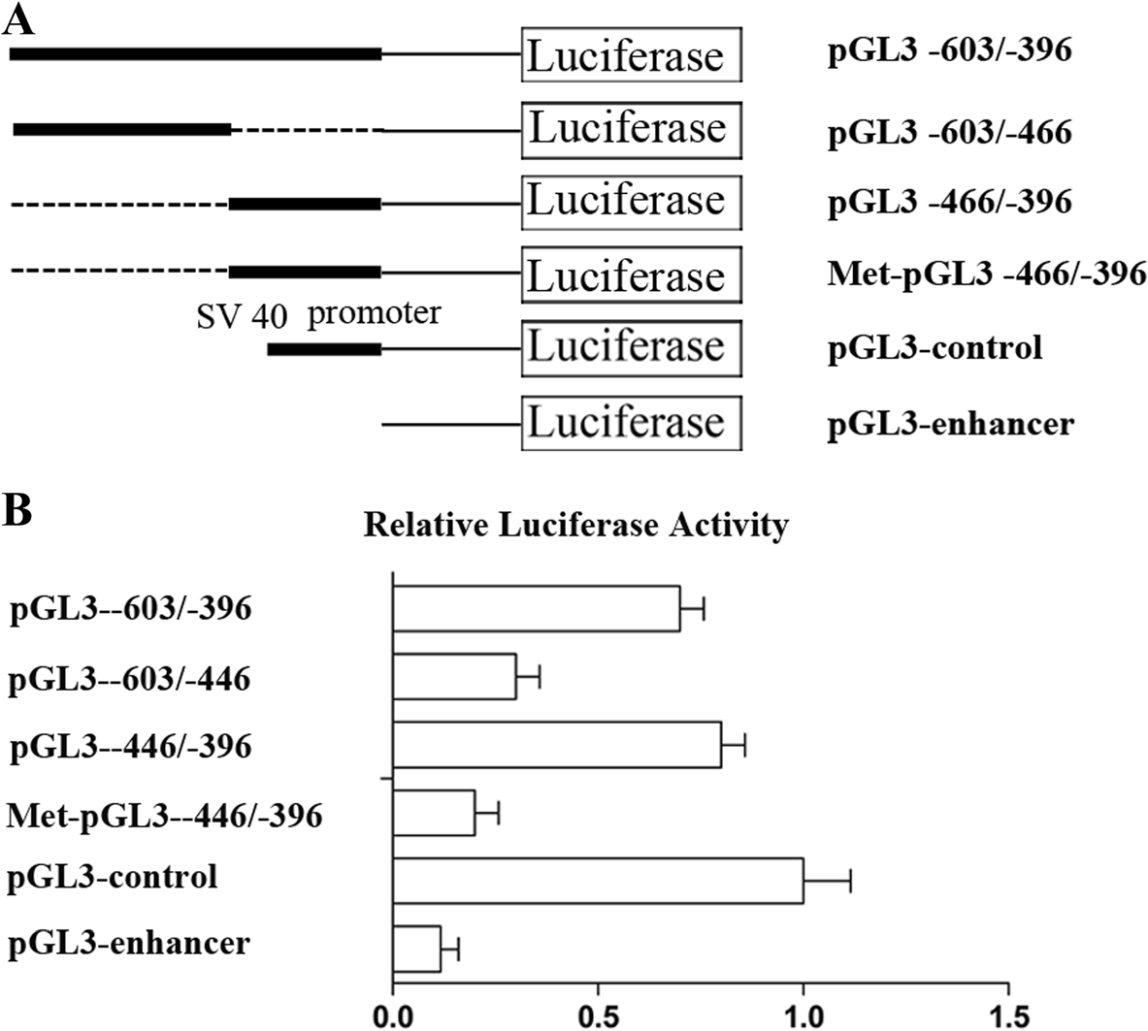
Identification of *ERCC6* minimal promoter. **a** Schematic illustration of deletion constructs of *ERCC6* proximal promoter pGL3 -603/-396, Met-pGL3 -466/-396, pGL3 control, and pGL3 enhancer. **b** Luciferase activity of the deleted constructs in 293T cells. Data are expressed as mean ± SD of three independent experiments. The *thick lines* represent retained parts of overall length (from -466 to -396, relative to the TSS). The *dash lines* represent the removed parts of overall length. The *thin lines* only represent the link to plasmid. Met-pGL3 -466/-396 represents methylated pGL3 -466/-396 without changing the sequences

In order to define the minimal promoter of *ERCC6* gene, three progressive 5′ ends deletion constructs (pGL3 -603/-396, pGL3 -603/-446, and pGL3 -446/-396) and Met-pGL3-446/-396 were generated (Fig. 3a). The vectors were transiently transfected into 293T cell lines. Luciferase expression levels were corrected for variable transfection efficiencies by co-transfection with a β-galactosidase plasmid. As shown in Fig. 3b, pGL3 -603/-446, pGL3-446/-396, Met-pGL3-446/-396, pGL3-control, and pGL3-enhancer had different luciferase expressions. Luciferase expression in Met-pGL3-446/-396 and pGL3 -603/-446 transfected cells was extremely low. These results indicated that the fragment spanning positions from -446 to -396 bp (a putative Sp1 binding site located in the region) is essential for the basal transcriptional activity of the *ERCC6* promoter.

### Expression of DNMTs in the LECs of controls and ARNCs

**Fig. 4:**
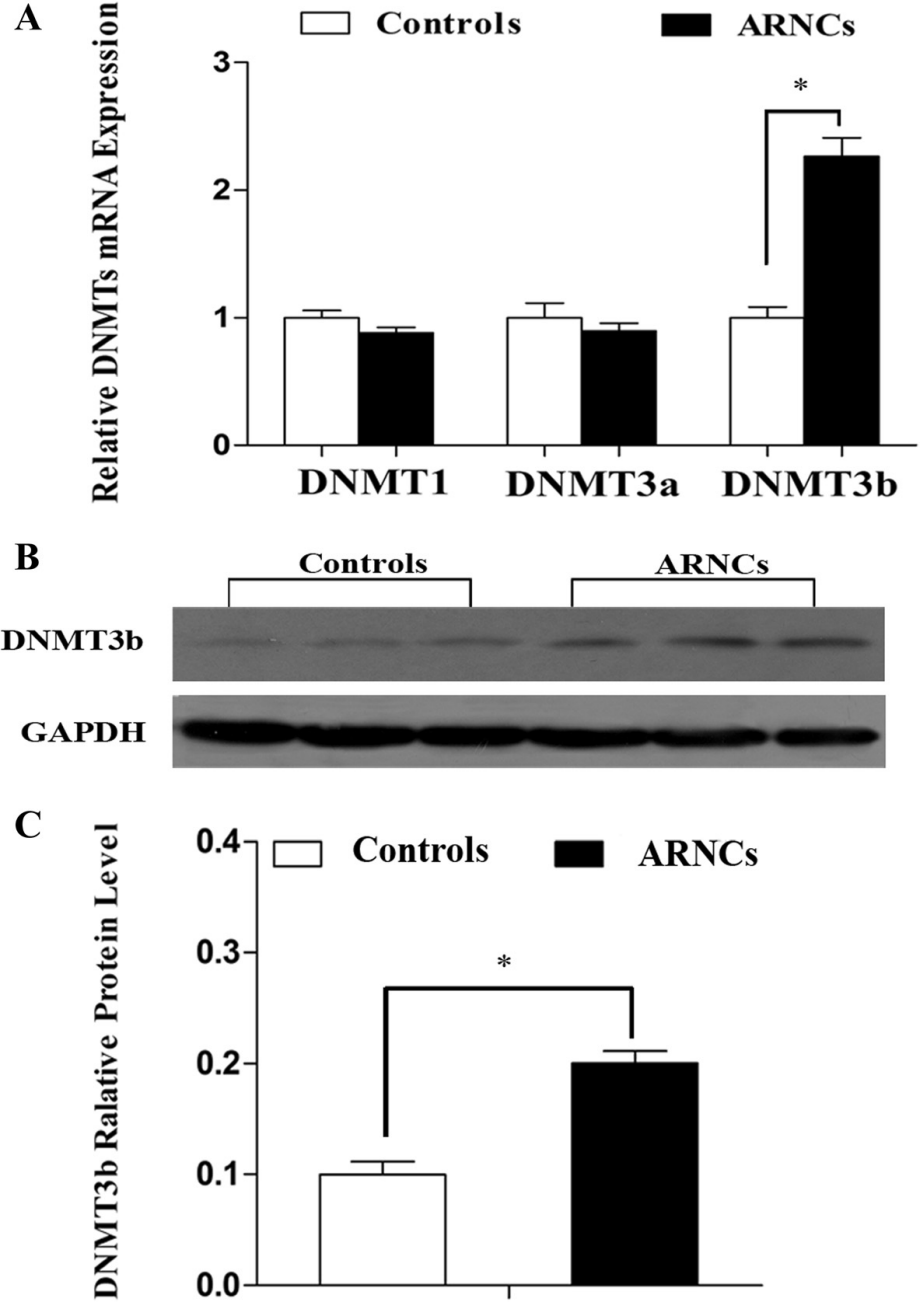
Relative expression of mRNA and protein level of *DNMTs* in LECs of controls and ARNCs. **a** qRT-PCR analysis of the mRNA expression of *DNMTs* in LECs of controls and ARNCs. The mRNA levels were normalized by using the GAPDH as the inner control. Values represent mean ± SD. *P < 0.01. **b** Western bolt analysis for protein level of DNMT3b in LECs of controls and ARNCs (Samples labeled as #1–#15 of the controls and #1–#15 of the ARNCs out of total 30 samples in each group). **c** Relative DNMT3b protein level to GAPDH is presented as mean ± SD. *P < 0.01

As DNA methylation is catalyzed and maintained by DNA methyltransferases DNMTs, we investigated the mRNA expression of DNMTs and found that DNMT3b was expressed at a 2.38-fold higher in LECs of ARNCs than that of the controls (Fig. 4a). Higher protein levels of DNMT3b were also detected in LECs of ARNCs than the controls (Fig. 4b, c). However, we did not observe differentiated expression for *DNMT1* and *DNMT3a* in LECs between the controls and ARNCs.

### Expression and distribution changes of ERCC6 and DNMT3b immunoreactivity in LECs of controls and ARNCs

**Fig. 5:**
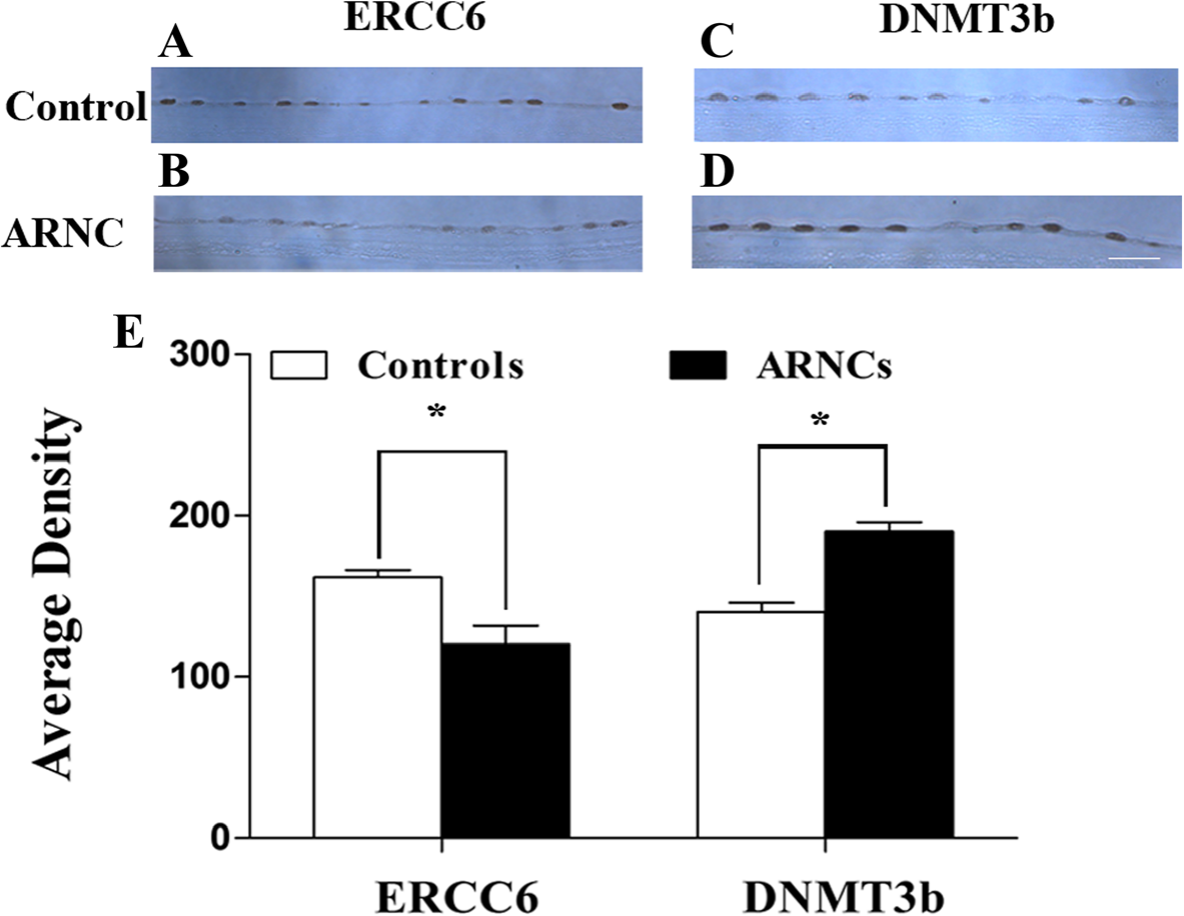
The staining changes of ERCC6 and DNMT3b immunoreactivity in LECs of controls and ARNCs (Samples labeled as #16–#30 of the controls and #16–#30 of the ARNCs out of total 30 samples in each group). The LECs of controls showed low levels of DNMT3b and high levels of ERCC6 staining (**a**, **c**). DNMT3b immunoreactivity increased significantly and the ERCC6 immunoreactivity decreased significantly in LECs of ARNCs (**b**, **d**). *Bar graph* illustrated the immunoreactivity of ERCC6 and DNMT3b in LECs of controls and ARNCs (**e**). *P < 0.01. *Scale bars*: 50 μm

To identify the distribution and expression changes of ERCC6 and DNMT3b in LECs, immunohistochemistry was performed with anti-ERCC6 and anti-DNMT3b monoclonal antibodies on LECs. ERCC6 staining was weakly expressed in LECs of ARNCs. There were high levels of ERCC6 staining in LECs of controls (Fig. 5a, b). In contrast, DNMT3b staining was weakly expressed in LECs of controls and high levels in LECs of ARNCs (Fig. 5c, d). These results showed that ERCC6 and DNMT3b expression were correlated with Western blotting and mRNA data in LECs (*P < 0.01; Fig. 5e).

### UVB reduced the expression of *ERCC6* and induced apoptosis and methylation at the CpG site 8 of *ERCC6* promoter in HLE-B3

**Fig. 6:**
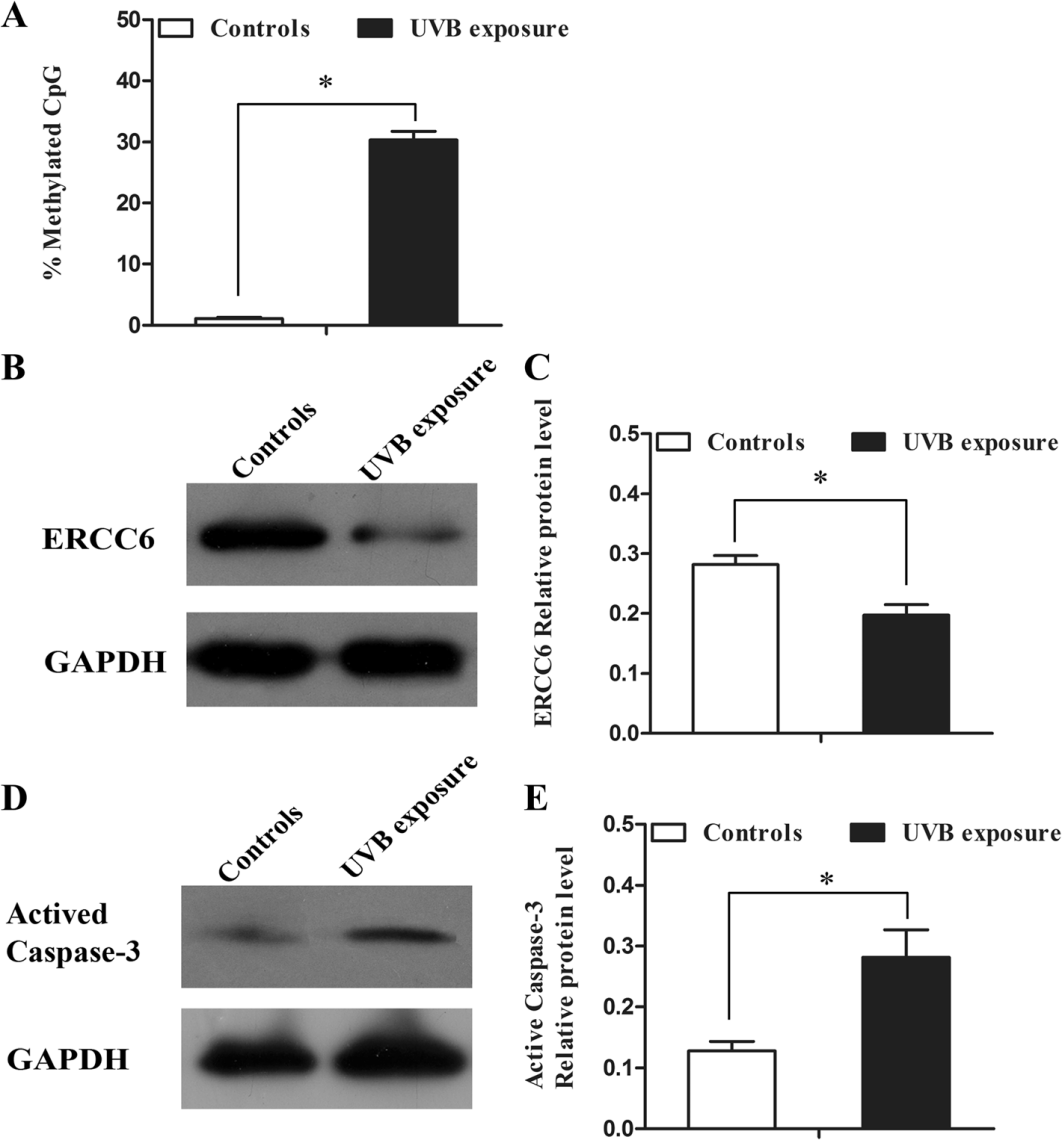
Protein level and promoter methylation status (site 8) of *ERCC6* in HLE-B3 after treatment with UVB exposure. **a** 12 h after treatment with UVB exposure, the promoter of *ERCC6* in the cells displayed hypermethylation compared to the control cells. *P < 0.01. **b** Protein levels of ERCC6 in control cells and in cells after treatment with UVB exposure. **c** Relative ERCC6 protein level to GAPDH is presented as mean ± SD. *P < 0.01. **d** Protein level of active caspases-3 in control cells and HLE-B3 cells after expose to UVB was measured by Western bolt analysis. **e** Relative active caspases-3 protein level to GAPDH is presented as mean ± SD. *P < 0.01

We determined the methylation status of *ERCC6* promoter in HLE-B3 treated with UVB exposure for 12 h. As shown in Fig. 6a, the methylation rate of CpG site 8 was 30.7 % in the UVB exposed cells and was 1.5 % in non-exposed cells. In the UVB radiation HLE-B3 cells, the expression of *ERCC6* was lower than that of control cells (Fig. 6b, c). Moreover, under these conditions, caspase-3 was activated to a larger extent in UVB-irradiated than non-irradiated cells, suggesting impaired repair of UVB-induced DNA damage in the cells (Fig. 6d, e).

### Suppression of cellular methylation status restored the ERCC6 expression

**Fig. 7:**
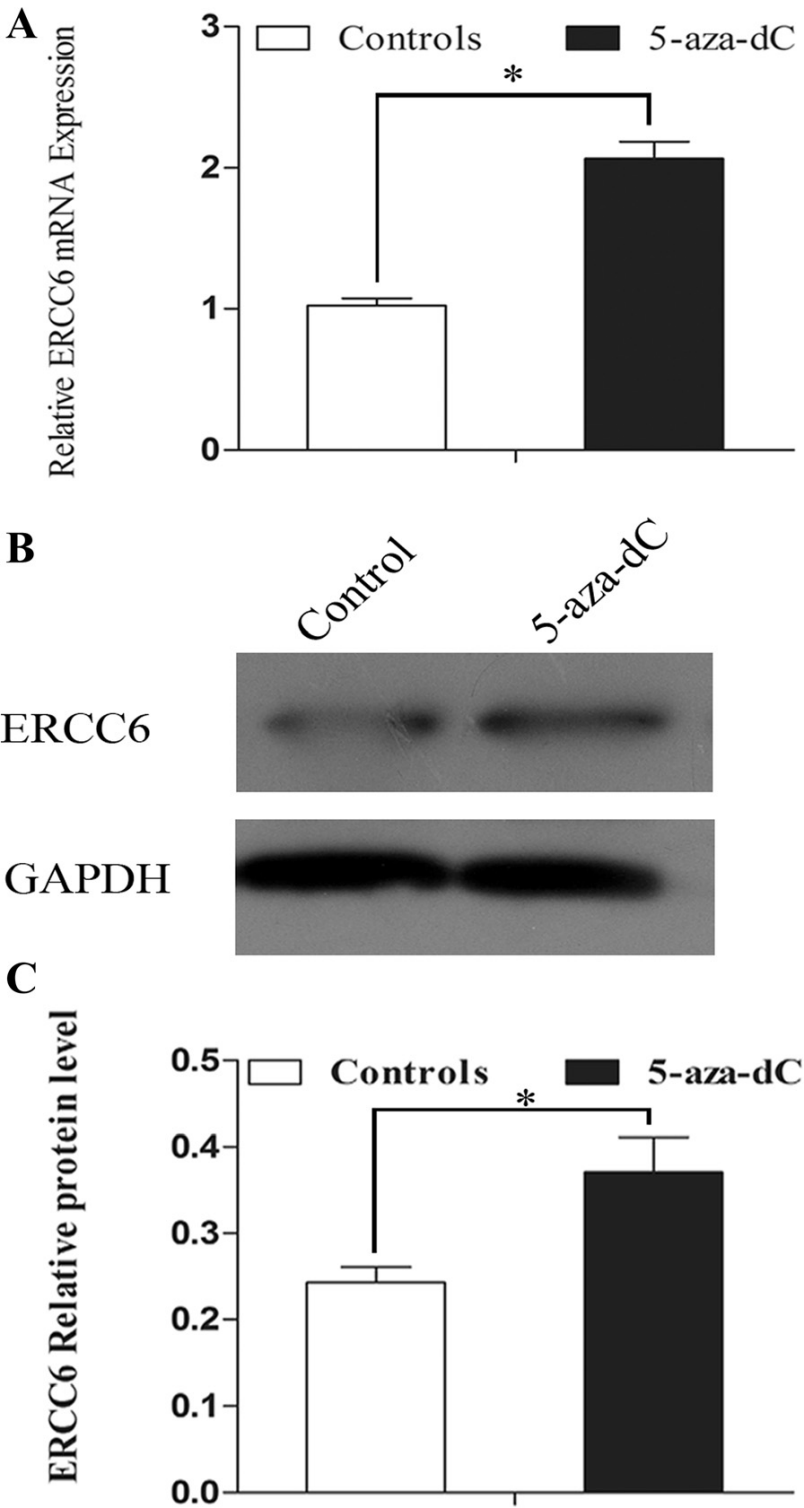
Relative mRNA expression and protein levels of *ERCC6* in HLE-B3 after treatment with 5-aza-dC. **a** mRNA expression of *ERCC6* in control cells and in cells after treatment with 5-aza-dC for 48 h. **b** Protein level of *ERCC6* in control cells and in cells after treatment with 5-aza-dC. **c** Relative ERCC6 protein level to GAPDH is presented as mean ± SD. *P < 0.01

To investigate the role of the methylation status in the transcriptional regulation of the *ERCC6* gene, we examined the effects of DNA methyltransferase inhibitor 5-aza-dC on *ERCC6* at the mRNA and protein level in HLE-B3 after exposure to UVB. We found that 5-aza-dC increased mRNA expression (Fig. 7a) and protein level (Fig. 7b, c) of *ERCC6*. These results suggest that the suppression of methylation process can upregulate *ERCC6* gene expression.

### The methylation of the *ERCC6* promoter at a CpG site abrogated the binding of Sp1

**Fig. 8:**
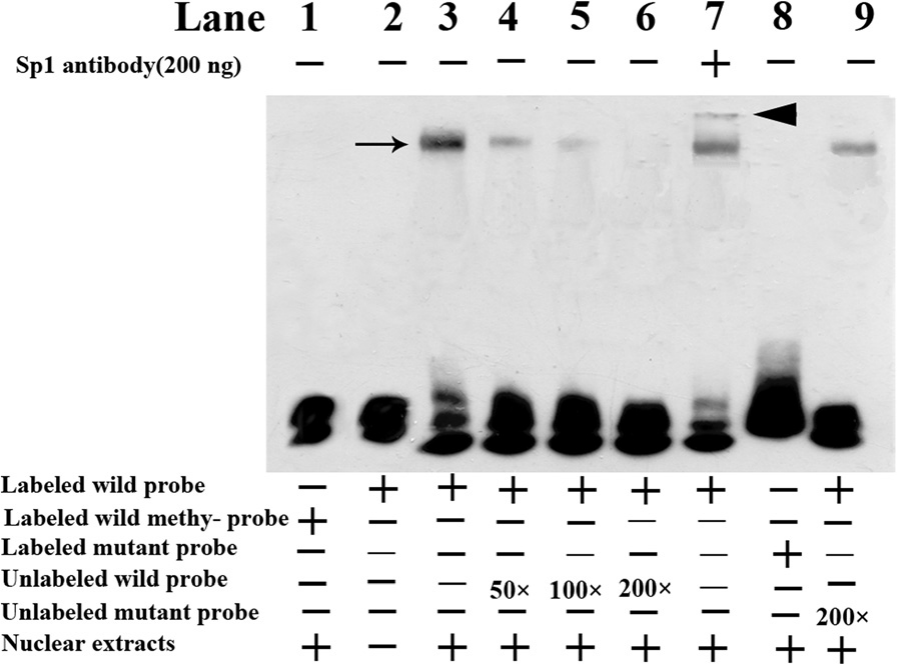
Electrophoretic mobility shift detected the DNA-binding ability of Sp1 on the ERCC6 promoter. The DNA-protein complex (*indicated by black arrow*) can be observed (*lanes 3*, *4*, *5*, *7*, and *9*). In contrast, no nucleoprotein complex was observed when used the M-probe and labeled mutated type probe (*lanes 1* and *8*). The competition assay was done by the addition of 50-, 100-, or 200-fold molar excess of unlabeled methylated probes to the incubation mixtures (lanes 4, 5, and 6). The complex formation was fully suppressed by the addition of a 200-fold molar excess of unlabeled wild type probe (lane 6) and not was suppressed by 200-fold molar excess of unlabeled mutated type probe (lane 9). A supershift band (indicated by *black arrowhead*, lane 7) was detected when anti-Sp1 antibody was incubated

Since the CpG site 8 was a potential transcription factor Sp1 binding site, we performed EMSA (electrophoretic mobility shift assay) with an unmethylated (UnM) or a methylated (M) probe spanning the Sp1 active site of the *ERCC6* promoter. A strong complex was observed when nuclear extracts from HLE-B3 cells were incubated with UnM-probe (Fig. 8a, lane 3). The band was supershifted by the antibody against Sp1 (Fig. 8a, lane 7). In contrast, no nucleoprotein complex was observed when the M-probe was used (Fig. 8a, lane 1). The complex formation was fully suppressed by the addition of a 200-fold molar excess of unlabeled wild type oligonucleotide probe (Fig. 8a, lane 6). This suppression was not observed when 200-fold molar excess of unlabeled mutated type oligonucleotide probe was added as competitor (Fig. 8, lane 9). These results further showed that transcription factor Sp1 specifically bind to the region of *ERCC6* promoter and methylation of the CpG site 8 could abrogate the binding of Sp1 to the region.

### Requirement of Sp1 for induction of *ERCC6* expression

**Fig. 9:**
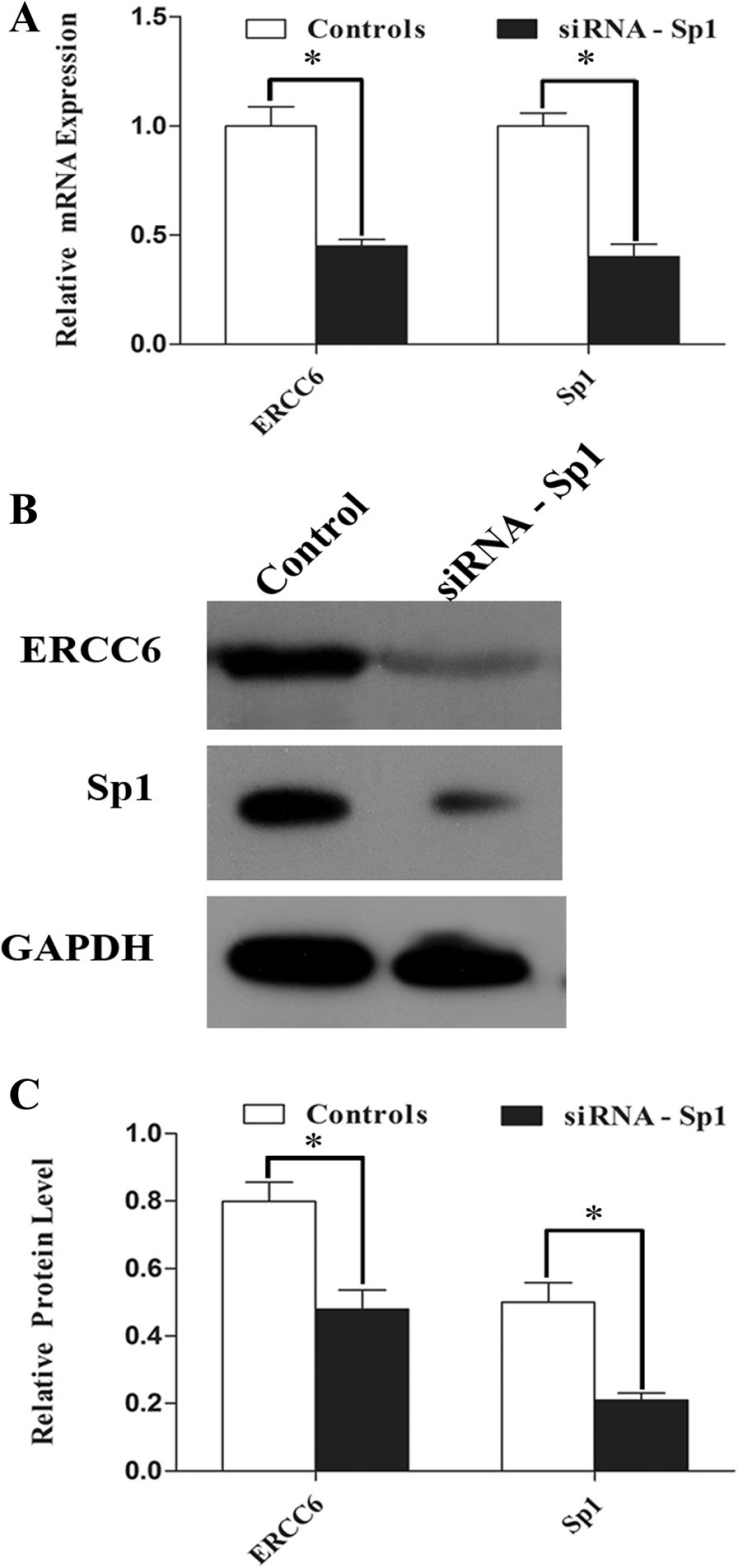
Knockdown of endogenous Sp1 decreased the mRNA expression and protein level of *ERCC6* and Sp1 in 239T cells. **a** Detection of *ERCC6* and Sp1 mRNA expression by qRT-PCR. Values represent mean ± SD. *P < 0.01. **b** Protein level of *ERCC6* and Sp1 was examined by Western blot analysis. Results are presented as mean ± SD. **c** Relative ERCC6 protein level to GAPDH is presented as mean ± SD. *P < 0.01

To investigate the role of endogenous Sp1 in regulation of *ERCC6* expression, siRNA technology for silencing Sp1 was used. The 293T cells were transfected with siRNA-Sp1 or siRNA-control, respectively. Total mRNA andprotein were isolated from harvested cells after 48-h transfection. qRT-PCR (Fig. 9a) and Western blot assay (Fig. 9b, c) revealed that compared with siRNA-control, transfection with siRNA-Sp1 reduced the expression levels *ERCC6* and Sp1. Taken together, these results indicated that Sp1 is required for *ERCC6* expression.

### UVB reduced the binding of Sp1 to *ERCC6* promoter and altered epigenetic modification at *ERCC6* promoter region

**Fig. 10:**
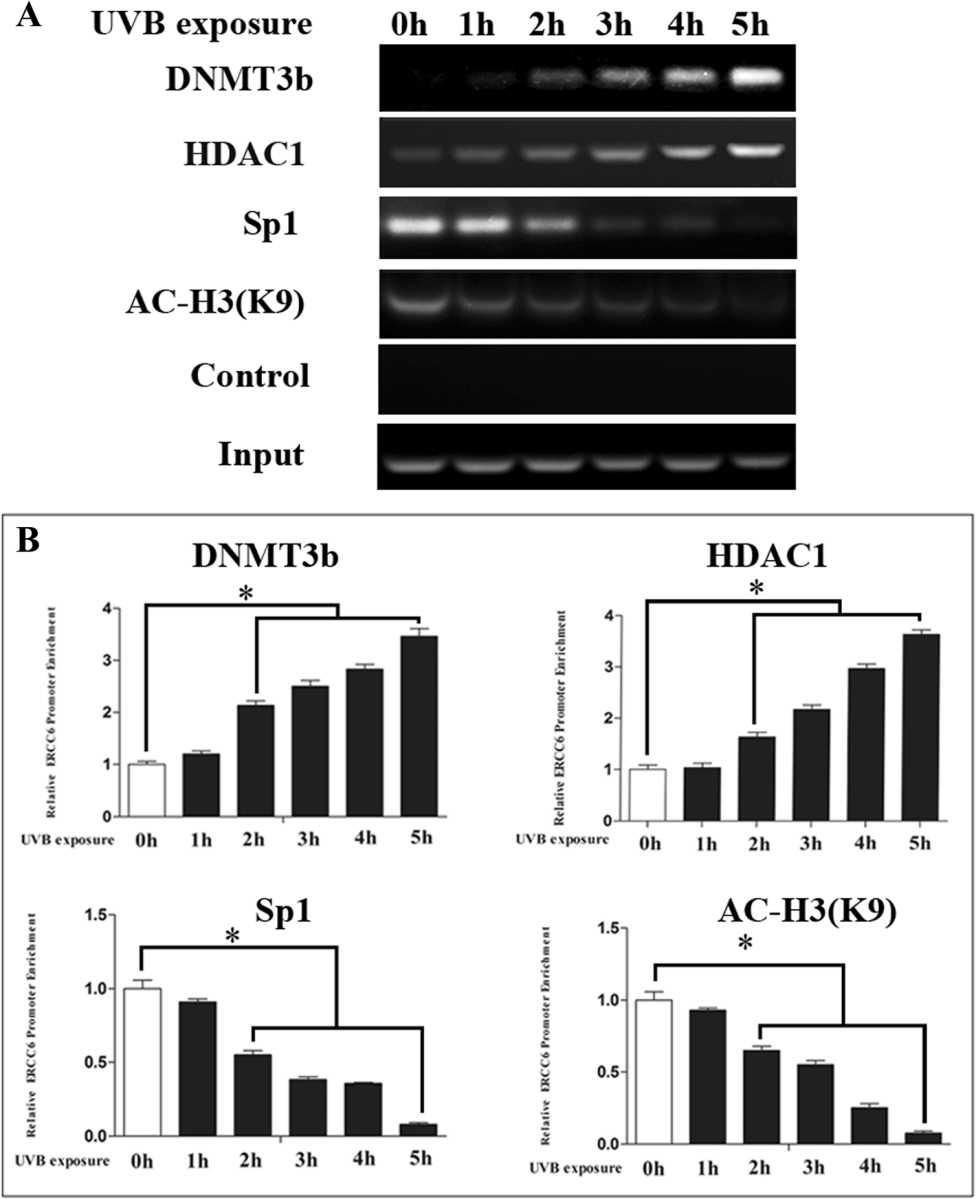
UVB irradiation decreased the occupancy of endogenous Sp1 and H3K9 acetylation on the *ERCC6* promoter, but increased occupancy of DNMT3b and HDAC1 on the *ERCC6* promoter. **a** HLE-B3 cells were treated with UVB irradiation. ChIP was performed using anti-Sp1, anti-DNMT3b, anti-HDAC1, and anti-ac-H3 (K9). **b** Statistically significant differences from control cells were indicated by *(*P* < 0.01)

A CHIP assay was performed to investigate whether transcription factor Sp1 interacted with *ERCC6* promoter through their specific binding sites in vivo. The DNA samples were immunoprecipitated in HLE-B3 cells by monoclonal antibodies against Sp1, and a DNA fragment of *ERCC6* promoter could be amplified by PCR. As shown in Fig. 10a, b, Sp1 binding to the *ERCC6* promoter was very strong in the cells without UV exposure, whereas the Sp1 binding to the *ERCC6* promoter became weak after UVB exposure for 5 h. The finding indicated that UVB exposure decreased the occupancy of endogenous Sp1 on *ERCC6* promoter. We then investigated whether DNMT3b recruitment via association with Sp1 works in coordination with DNA methylation during UVB exposure. We found the amount of DNMT3b that occupied the *ERCC6* promoter was strong after treated with UVB exposure. As shown in Fig. 10a, deacetylated H3K9 was significantly increased after UVB exposure which was consistent with the amount of occupancy of HDAC1 on *ERCC6* promoter. These findings indicated that UVB exposure reduced the recruitment of Sp1 and induced the recruitment of DNMT3b and HDAC1 at the *ERCC6* promoter as well as the hypermethylation of CpG site 8 and an increased H3K9 deacetylation.

### MS275 increase *ERCC6* gene transcription

**Fig. 11:**
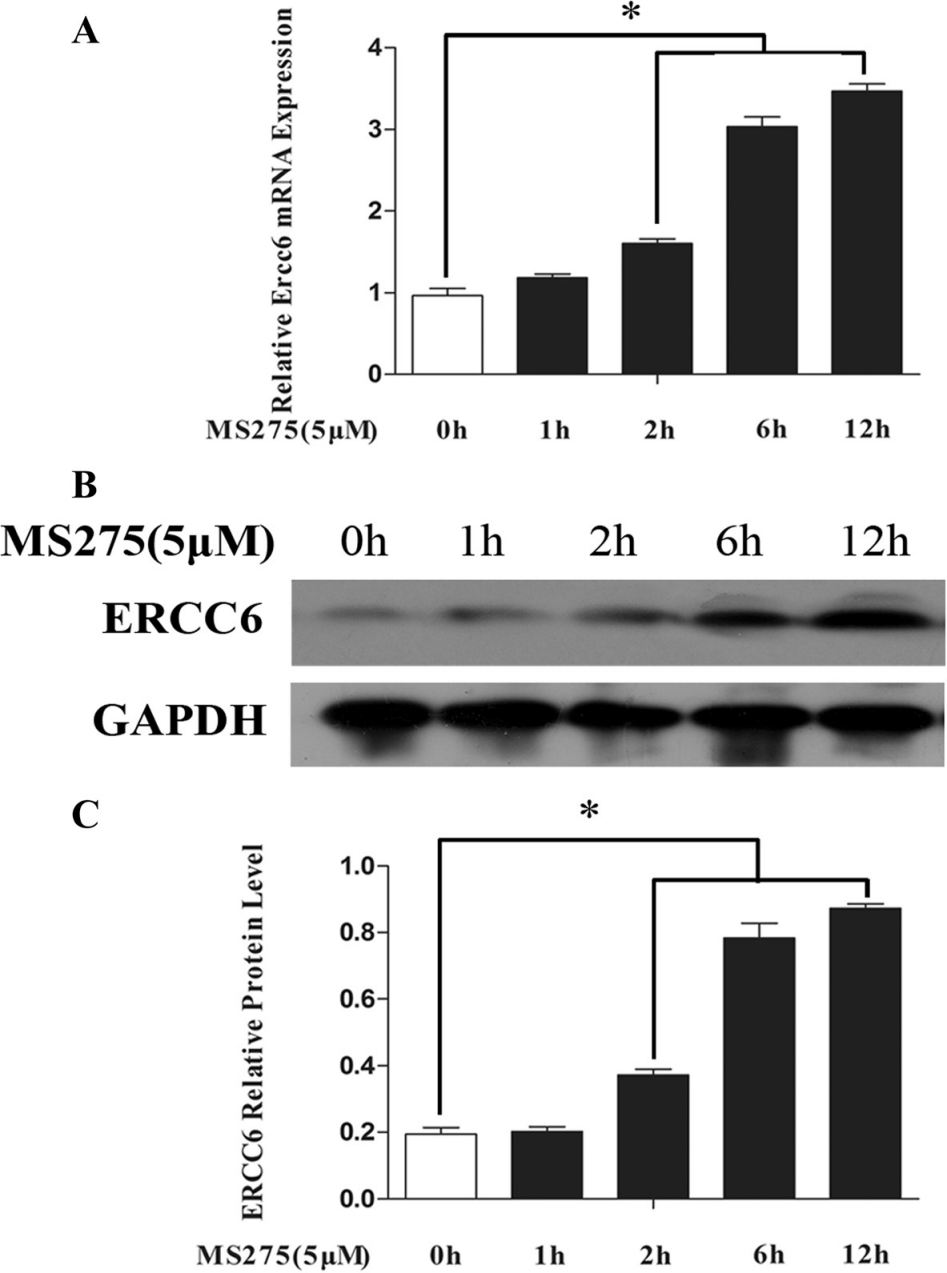
Relative mRNA expression and protein levels of *ERCC6* in HLE-B3 after treatment with MS275. **a** Detection of *ERCC6* mRNA expression by qRT-PCR at different time point. **b** Protein level of *ERCC6* was examined by Western blot analysis at different time point. **c** Relative ERCC6 protein level to GAPDH is presented as mean ± SD. *P < 0.01

In order to verify whether *ERCC6* gene expression was regulated by histone acetylation state, we treated HLE-B3 cells with MS275 (histone deacetylase inhibitor) and then measured *ERCC6* mRNA and protein levels at different time points (from 1 to 12 h). As showed in Fig. 11, MS275 induced *ERCC6* mRNA levels and protein expression, starting at 1 h after the treatment in HLE-B3 cells.

## Discussion

In recent years, some reports including our studies have shown the important role of epigenetic modifications in pathogenesis of cataract [26, 40–42]. Patterns and levels of DNA methylation and histone modification are the most studied epigenetic modifications in the context of gene transcription. In this study, overexpression of *DNMT3b* was correlated with DNA hypermethylation status of *ERCC6* promoter in LECs of ARNCs. Using promoter deletion constructs, we found that the -446 to -396 (relative to TSS) region of *ERCC6* gene is the crucial regulatory region for *ERCC6* expression. We identified that hypermethylation at a CpG site blocked Sp1 binding in the region, thereby suppressing *ERCC6* expression. In HLE-B3, exposure to UVB strongly induced the DNA hypermethylation at the CpG site in promoter of *ERCC6* and deacetylated of H3K9. Then, treatment with 5-aza-dC and MS275 can restore the *ERCC6* expression. We also showed that caspase-3 was activated at a higher extent in HLE-B3 cells exposed to UVB compared to non-irradiated controls, suggesting reduced repair of the UV damage, as expected. These data nevertheless underscore the link between epigenetic modifications at the ERCC6 site and reduced DNA repair. 

Identifying specific methylated sites is crucial for understanding the mechanisms behind regulating gene expression, as it is known that the interaction between single CpG site and hypermethylation can be sufficient to adjust gene transcription [43]. We reported that the hypermethylation in the promoter of *MGMT* can reduce the expression of the gene and altered expression of *MGMT* is associated with the pathogenesis of ARCs [40]. Others also reported that the methylation status of a critical CpG site was associated with transcriptional inactivation of the p53 gene [44]. A single base pair or epigenetic changes in DNA sequence can alter protein binding. For example, a single nucleotide polymorphism (SNP) in 5′ flanking region of *ERCC6* affects the binding of Sp1 [45, 46]. In the present study, hypermethylation at a CpG site located within the Sp1 binding sequence on the *ERCC6* promoter was found to be associated with Sp1 binding. Similar findings were reported in the Sp1 binding elements of several other genes [47–49]. Our results showed an increased DNMT3b binding to the region with an induced hypermethylated at a CpG site of *ERCC6* when HLE-B3 exposed to UVB. Some studies showed exposure to UVB-enhanced expression of DNMT1, DNMT3a, and DNMT3b as well as DNMT activity, which subsequently increased global DNA methylation [38, 39]. DNMT3b, unlike DNMT1, is known to act in de novo methylation, targeting unmethylated CpG sites [50]. DNMT3b overexpression can significantly induce the global DNA hypermethylation in a recent research [51]. But in this study, we only focused on the hypermethylation of special sites in *ERCC6* promoter and cannot exclude possible changes of global methylation.

Several reports implied that DNA hypermethylation could be triggered by higher levels of histone deacetylation [52, 53]. A study showed that HDAC1 is recruited to the Sp1 binding site at the CpG island of LEDGF gene in LECs [54]. It has been reported that significant deacetylations at H3K9 are specifically regulated by HDAC1 [55]. So we elucidated a more complex epigenetic role on *ERCC6* expression by treatment HLE-B3 with UVB exposure. We showed that HDAC1 association at the *ERCC6* promoter was significantly induced after expose to UVB and deacetylation of H3K9 increased in this region. The restoration of *ERCC6* expression suggests that inhibition of HDAC1 is sufficient and necessary to activate *ERCC6* promoter. The findings shown here support a model of chromatin structure changes in HLE-B3 when expose to UVB. Specific CpG hypermethylation followed by H3K9 deacetylations reduced binding of Sp1 at *ERCC6* gene regulatory region and a suppressed transcription of *ERCC6*. Collectively, current data indicate that Sp1 binding site in the region is under dynamical epigenetic reprogramming (methylation and deacetylation of histones) in HLE-B3 facing UVB exposure.

## Conclusions

We found that *ERCC6* transcription may be epigenetically regulated in LECs of ARNCs, leading to *ERCC6* repression. Results reported here provide a novel gene repression mechanism, emerging from integrated changes in the levels of cytosine hypermethylation and histone deacetylation in a special CpG site, which control *ERCC6* transcription. Our study does not exclude that other cofactors can be recruited to the* ERCC6* locus and be involved in the control of *ERCC6* expression. The finding of epigenetic factors in LECs of ARNCs might provide a proof of concept for the intervention of DNA methylation and histone modification in ARNCs therapy and prevention.

## Methods

### Study participants

According to the Lens Opacities Classification System III (LOCS III) [56], we selected 30 patients with ARNCs and 30 age-matched controls who had their transparent lens extracted because of vitreoretinal diseases in this study. We excluded patients with complicated cataracts due to high myopia, uveitis, ocular trauma, or other known causes; other major eye diseases such as glaucoma, myopia, diabetic retinopathy, and uveitis; and systematic diseases such as hypertension and diabetes. The basic demographic of the study participant was listed in the Table 1. All procedures conformed to the Declaration of Helsinki, and written informed consent was acquired from all participants. The consent procedure was also approved by the ethics committee of Affiliated Hospital of Nantong University.

**Table 1:** Demographic data of the controls and ARNCs

	Controls	ARNCs	
	(*n* = 30)	(*n* = 30)	*P*
Sex			>0.05
Male, *n* (%)	13 (43.33 %)	14 (46.67 %)	
Female, *n* (%)	17 (56.67 %)	16 (53.33 %)	
Age, year; mean ± SD	68.52 ± 7.56	70.79 ± 6.31	>0.05

### Collection of lens anterior capsule membrane samples

The centered anterior capsules of lens from controls and ARNCs were carefully obtained by anterior continuous curvilinear capsulorhexis during cataract surgery. The samples were rapidly frozen in liquid nitrogen and stored at −80 °C.

### Cell culture and treatment with 5-aza-dC, MS275, and UVB radiation

Human lens epithelium B3 (HLE-B3) and 239T cell lines were obtained from American Type Culture Collection (ATCC; Rockville, MD) and cultured in Dulbecco’s modified Eagle’s medium (DMEM; Sigma-Aldrich) with 10 % (*v*/*v*) fetal bovine serum (FBS; Sigma) in a humidified atmosphere of 5 % CO_2_ at 37 °C. We used the 293T cell line because this cell line is a common model for vector transfection with high transfection efficiency. We used the HLE-B3 for other experiments because the cells are considered a possibility host for intervention and UV radiation experiment [57]. HLE-B3 was cultured in six-well plates in DMEM without FBS for 24 h prior to treatment. Then, the HLE-B3 were treated with 10 μM 5-aza-dC (Sigma, St. Louis, MO) for 48 h and 5 μM MS275 (Selleckchem, Houston, TX, USA) for 12 h, and exposed to UVB light for 20 min. We harvested the cells on different time point (1000 J/m^2^, XX-15B, Spectroline, Westbury, NY, USA), respectively. The intensity and dose of UBV were measured using a UVX Radiometer connected to a UVX-31 Sensor (both were from UVP Inc., San Gabriel, CA, USA). After exposure, the DMEM was immediately replaced by DMEM with 10 % FBS. At different time point, the cells were harvested for DNA, mRNA, and protein extraction.

### RNA extraction and reverse transcription

Total RNA was extracted from the treatment cells, no-treatment cells, and LECs using the Trizol reagent (Life Technologies Corporation, Carlsbad, CA, USA) according to the manufacturer’s recommendation. Then, 1-μg total RNA was subjected to reverse transcription with PrimeScript® RT reagent Kit (Takara, Dalian, China) according to the manufacturer’s instructions.

### Quantitative reverse-transcription polymerase chain reaction (qRT-PCR)

TaqMan gene expression assay probes (Applied Biosystems, Foster City, CA) were used for *ERCC6*, *DNMT1*, *DNMT3a*, *DNMT3b*, and *Sp1* mRNA quantification (assay ID: Hs00972920_ml, Hs00945875_ml, Hs01027166_ml, Hs00916521_m1, and Hs00171876_ml). *GAPDH* (Hs99999905_m1) was used as an internal control. qRT-PCR was performed using the ABI 7500 Real-Time PCR System (Applied Biosystems). The fold change of gene expression was determined using the comparative CT method (2^−ΔΔCT^), and each sample was analyzed in triplicate.

### Western blot assay

Lysates of LECs and cultured cells were prepared for Western blot analysis as described previously [26]. After the determination of its protein concentration with the Bradford assay (Bio-Rad, USA), samples with equal amounts of protein was subjected to SDS-polyacrylamide gel electrophoresis (PAGE) (100 V for 90 min) and transferred to a polyvinylidine difluoride membrane (Millipore, Bedford, MA) by a transfer apparatus (Bio-Rad) at 40 mA for 8 h. Nonspecific protein binding to the membrane was blocked with blocking buffer (5 % nonfat milk, 200 mM NaCl, 50 mM Tris, 0.05 % Tween 20). The blocked membrane was then incubated with primary antibodies rabbit anti-human-ERCC6 (1:1000, Abcam, Inc., Cambridge, MA, USA), mouse anti-human-Sp1 (1:1000, Millipore, Billerica, MA), goat anti-human-DNMT3b (1:1000, Abcam), mouse anti-human active caspase-3 (1:1000; Abcam), and rabbit anti-human-GAPDH (1:2000; Abcam) at 4 °C for 12 h. After the membrane was washed three times with TBST (20 mM Tris, 500 mM NaCl, 0.1 % Tween 20) for 5 min each time at 28 °C, the membrane was incubated withalkaline phosphatase-conjugated secondary antibodies (1:4000; Santa Cruz, USA) for 2 h at 28 °C. Then, the membrane was washed four times with TBST for 15 min each time at 28 °C. Detection was performed using an ECL chemiluminescence kit (Pierce, Rockford, IL). The film was scanned using ImageQuant software (Molecular Dynamics, Sunnyvale, CA). The gray value of each protein band was measured, and data are presented as a ratio of this value to that for GAPDH.

### Immunohistochemistry

The centered anterior capsules were fixed with 4 % paraformaldehyde for 3 days and embedded in paraffin. Before staining, 4-μm-thick sections were cut for immunohistochemistry. After being washed, sections were incubated with rabbit anti-human-ERCC6 (1:200; Abcam) and goat anti-human-DNMT3b antibodies (1:200; Abcam) for 12 h at 4 °C. Then, sections were incubated in biotinylated secondary antibody (Vector Laboratories, Burlingame, CA, USA), followed by incubation in the complex avidin-biotin-peroxidase (ABC Kit, Vector Laboratories, Burlingame, CA, USA). Staining was visualized with DAB (Vector Laboratories).

The staining signal was quantitated using Image-ProH Plus 6.0 software (Media Cybernetics, Inc., Bethesda, MD, USA) as previously described [42]. Anterior capsule membrane sections from patients of controls and ARNCs were used in each experiment. The staining signal in each section was measured in at least five different fields. The total integrated optical density (IOD) of the area of interest (AOI) in each field was recorded. Data are presented as the mean density of the immunostaining area.

### Bioinformatic analysis

The genomic DNA sequences of *ERCC6* were downloaded from the NCBI genome database. Transcription start site (TSS) was predicted by the online database (http://genome.ucsc.edu/). The CpG islands of the promoter region were predicted by online software (http://www.urogene.org/cgi-bin/methprimer/methprimer.cgi), with the setting of the confidence intervals, minimum CG content >50 %, ratio between observed and expected CpG-0.6, and minimum CpG island length 100 bp. Potential binding sites of transcription factors within the CpG island of *ERCC6* gene were analyzed by the online software (http://alggen.lsi.upc.es/cgi-bin/promo_v3/promo/promoinit.cgi?dirDB=TF_8.3).

### Isolation of genomic DNA, sodium bisulfite conversion, and methylation assays by pyrosequencing

The isolation of genomic DNA from the cells and LECs was performed by standard phenol–chloroform extraction. Two micrograms of genomic DNA were treated with sodium bisulfite using the EpiTect Bisulfite Kit (Qiagen, Inc., Frederick, MD). Quantitative DNA methylation analysis of the bisulphite-treated DNA was performed by pyrosequencing [42]. Regions of interest were amplified using 20 ng of bisulfite-treated genomic DNA and 5 pmol of forward and reverse primer, one of them being biotinylated. Primers for PCR amplification and pyrosequencing were used to cover regions of interest (Table 2). In a 50-μl mixture that contained standard reaction conditions were HotStar Taq buffer supplemented with 25 mM MgCl_2_, 10 mM dNTPs, and 5.0 U HotStar Taq polymerase (Qiagen, MD USA). The PCR program consisted of a denaturing step of 3 min at 95 °C followed by 35 cycles of 30 s at 94 °C, 30 s at the respective annealing temperature, and 25 s at 60 °C, with a final extension of 5 min at 72 °C. After verification by standard gel electrophoresis on a 1 % agarose gel, 5 μl of PCR product was incubated, for 10 min at 28 °C with shaking, in the presence of 40 μl of binding buffer (10 mM Tris, 2 M NaCl, 1 mM EDTA, 0.1 % Tween 20; pH 7.6; adjusted with 1 M HCl), 2 μl of streptavidin-coated sepharose beads (Qiagen, MD USA), and 33 μl of ddH2O. The binding mix was purified and rendered single stranded using the Vacuum Prep Workstation (Qiagen, MD, USA) according to the manufacturer’s instructions. Beads were released into 12-μl annealing buffer (20 mM Tris, 2 mM Mg-acetate; pH 7.6; adjusted with 4 M acetic acid) containing 4 pmol of the respective sequencing primers. Primers were annealed to the target by incubation at 80 °C for 2 min. Quantitative DNA methylation analysis was carried out on a PyroMark Q96 ID pyrosequencer (Qiagen, MD USA) with the PyroMark Gold Q96 Reagent (Qiagen, MD, USA). For each locus, methylation status was analyzed individually as a T/C single nucleotide polymorphism using PyroMark CpG Software 1.0.11 (Qiagen, MD, USA).

**Table 2:** Primer sequences for pyrosequencing

Forward primer (5′-3′)	Reverse primer (5′-3′)	Sequence primer (3′-5′)	Product size (bp)
Biotin—GYGGGGAAGGGAGGAGTTTTA	CCCRTTCTCCRTCCCTTACCT	CCTACTTTAAAATTCAAAACCA	160
		TTCTCCTTCCCTTACCTCC	
Biotin—TGTGGAGTYGYGGAGGTAAGA	AAAACTTTATACCCRACATAAAAAA	CTTTATACCCTACATAAAAAAC	97
Biotin—TGTGGAGTYGYGGAGGTAAGGA	ACTCTACCRTTAAAACRACACTCACCC	ACATAATAACTAACTTCCTAACC	218

### Luciferase reporter vectors and assay

Three promoter plasmids (pGL3 -603/-396, pGL3 -603/-446, and pGL3 -446/-396 relative to the TSS) were constructed by Sangon Biotech (Shanghai, China) Co., Ltd. The Sp1 oligo small interfering RNA (siRNA) and a negative control siRNA were purchased from Santa Cruz Biotechnology, Inc. Cells were cultured in six-well plates at a density of 1 × 10^6^ cells/per well for 24 h prior to transfection in DMEM without FBS. The cells were transfected with 0.5 μg of various *ERCC6* promoter constructs, Sp1-siRNA(50 nM), a negative control siRNA(50 nM), pGL3-control (50 nM), and/or pGL3-enhancer plasmid (50 nM) using lipofectamine 2000 transfection reagent (Invitrogen, Germany) according to the manufacturer’s instructions. To control transfection efficiency, cells were co-transfected with 0.5 μg SV40 β-galactosidase vector per well. The cell lysates were prepared at 48 h after transfection, and luciferase activity was measured by luciferase assay kit (Promega). β-galactosidase activity was also quantified using the β-galactosidase Enzyme Assay System (Promega). Experiments were repeated at least three times with three replicates per sample.

### Electrophoretic mobility shift assay (EMSA)

Nuclear extracts from HLE-B3 cells were prepared with NE-PER Nuclear and Cytoplasmic Extraction Reagents (Thermo Scientific, Waltham, MA, USA) and were subjected to EMSA using the LightShift Chemiluminescent kit (Thermo Scientific). The binding reaction mixture containing 5 μg of nuclear extract, 20 fmol of 5′ biotin-labeled oligonucleotide probes, 1 × binding buffer, 50 ng of poly(dI•dC), 2.5 % glycerol, 0.05 % Nonidet P-40, and 5 mM MgCl2 was incubated at room temperature for 20 min in a final volume of 20 μl. For supershift assays, nuclear extracts were incubated with 200 ng anti-Sp1 antibody (Millipore) at 28 °C (for 20 min) prior to probe addition. An unmethylated (UnM) or methylated (M) wild oligonucleotide encompassing the potential Sp1 binding site was used: 5′-CTCGAAACCCCGCCTACCTCTG-3′. The sequence of the mutated (underlined nucleotides) oligonucleotide was 5′-CTCGAAACCTTTCCTTTTTCTG-3′. The methylated oligonucleotide were prepared by incubating 1 μg of unmethylated probe with 10 units Hpall methyltransferase (New England Biolabs Inc.), 10 μl 1 × Hpall methyltransferase buffer, supplemented with 80 μM S-adenosylmentionine at 37 °C 1 h followed by 15 min at 65 °C to inactivate the methylase, and purified by polyacrylamide gel electrophoresis. For competition assays, a 50-, 100-, and 200-fold excess of unlabeled wild double-stranded oligonucleotides was incubated with the extracts at 28 °C for 20 min before the probe addition. Bound complexes were separated on 6.5 % native polyacrylamide gels in 0.5 × TBE. Then, the binding reactions were transferred to nylon membrane (Thermo Scientific) with the parameters: 380 mA, and 25 °C for 0.5 h and crosslinking was performed with a hand-held UV lamp equipped (Shanghai Guang Hao Analysis Instrument Co., Ltd.) with 254 nm bulbs for 10 min. Finally, the biotin-DNA was detected by chemiluminescence and visualized by Biospectrum® 510 Imaging System (Upland, CA, USA).

### In vitro DNA methylation and transient transfection

The methylated plasmids (Met-pGL3-446/-396) were generated by incubating 1 μg of plasmid DNA (pGL3 -446/-396) with 10 units Hpall methyltransferase in 10 μl 1 × Hpall methyltransferase buffer and 80 μM S-adenosylmethionine according to the manufacturer’s protocols (New England Biolabs, Inc.). Reactions were carried out at 37 °C 1 h followed by 15 min at 65 °C to inactivate the methylase, purified by polyacrylamide gel electrophoresis. The methylated plasmid DNA was transfected into 293T cell lines in parallel with the unmethylated pGL-446/-396, respectively. Luciferase activity was analyzed at 48 h after transfection.

### Chromatin immunoprecipitation (ChIP) Assay

ChIP assays were performed by using Magna ChIP TM A/G (Millipore Corporation, Temecula, CA), and the sheared chromatin samples were used for immunoprecipitation with 1 μg of mouse anti-human-Sp1 (Millipore) anti-acety H3K9 (Upstate Biotechnology), rabbit anti-human-HADC1(Millipore), and goat anti-human-DNMT3b (Millipore) antibodies, overnight at 4 °C. Immunocomplexes were subjected to cross-link reversal, extracted, and precipitated as described in the protocol. The eluted DNA and the aliquots of chromatin prior to immunoprecipitation (input) were amplified by RT-PCR. To detect the DNA sequence of the *ERCC6* gene promoter, where the CpG site 8 is located, we used the following primer set: forward, 5′-TGTTTTGAATTTTTGTGTGGATATTT-3′; reverse, 3′-ACTATCCTACTTCTCTATTCCCCCTC-5′. The PCR conditions were as follows: 95 °C for 3 min and 35 cycles of 94 °C for 25 s, 60 °C for 25 s, and 72 °C for 5 min. PCR products were separated by 2 % agarose gel containing GoldviewII (Beijing Solarbio Science Technology Co., Ltd). Bands were visualized by Biospectrum® 510 Imaging System (Upland).

### Statistical analysis

The one-way analysis of variance (ANOVA) test was performed to identify the differences among the groups. Differences were considered significant when the *P* value was <0.05. SPSS software (SPSS 17.0; SPSS, Inc., USA) was used for performing statistical analysis.

## Abbreviations

5-aza-dc, 5-aza-2′-deoxycytidine; ARNC, age-related nuclear cataract; ChIP, chromatin immunoprecipitation; CSB, Cockayne syndrome complementation group B; DMEM, Dulbecco’s modified Eagle’s medium; DNMTs, DNA methyltransferases; EMSA, electrophoretic mobility shift assay; FBS, fetal bovine serum; HDACs, histone deacetylase; HLE-B3, human lens epithelium B3; LECs, lens epithelial cells; NER, nucleotide excision repair; NER, nucleotide excision repair; qRT-PCR, quantitative reverse-transcription polymerase chain reaction; SNP, single nucleotide polymorphism; TSS, transcription start site; UVB, ultraviolet-B.

## Additional file

Additional file 1: Table S1. The grade of lens opacity and identification codes of Controls and ARNCs (DOCX 21 kb) Additional file 2: Figure S1. In LECs of ARNCs, the CpG site 8 displayed hypermethylation compared to the controls (n = 30, respectively) (TIF 1989 kb)
