# Supplementary material for: Ultraviolet-B induces ERCC6 repression in lens epithelium cells of age-related nuclear cataract through coordinated DNA hypermethylation and histone deacetylation
Source: Clin Epigenetics. 2016 May 26;8:62. doi: 10.1186/s13148-016-0229-y (PMC4880862; doi:10.1186/s13148-016-0229-y)
Supplement: Additional file 1: Table S1. — The grade of lens opacity and identification codes of Controls and ARNCs (DOCX 21 kb) [file 13148_2016_229_MOESM1_ESM.docx]

| Controls | | | |  | ARNCs | | | |
| --- | --- | --- | --- | --- | --- | --- | --- | --- |
| Samples | Sex | Age(y) | LOCSⅢ |  | Samples | Sex | Age(y) | LOCSⅢ |
| No.1 | male | 74 | NO0C1P0 |  | No.1 | male | 74 | NO3C0P0 |
| No.2 | male | 66 | NO0C0P0 |  | No.2 | female | 64 | NO4C0P0 |
| No.3 | female | 69 | NO0C0P1 |  | No.3 | female | 71 | NO2C0P0 |
| No.4 | female | 64 | NO0C1P0 |  | No.4 | male | 69 | NO5C0P0 |
| No.5 | female | 53 | NO0C0P0 |  | No.5 | female | 84 | NO4C0P0 |
| No.6 | male | 69 | NO0C0P1 |  | No.6 | female | 76 | NO3C0P0 |
| No.7 | female | 68 | NO0C1P0 |  | No.7 | male | 64 | NO5C0P0 |
| No.8 | male | 68 | NO0C0P0 |  | No.8 | female | 68 | NO4C0P0 |
| No.9 | female | 78 | NO0C1P0 |  | No.9 | male | 63 | NO5C0P0 |
| No.10 | male | 65 | NO1C0P0 |  | No.10 | female | 66 | NO3C0P0 |
| No.11 | female | 66 | NO0C0P1 |  | No.11 | female | 78 | NO2C0P0 |
| No.12 | female | 78 | NO1C0P0 |  | No.12 | female | 67 | NO5C0P0 |
| No.13 | male | 72 | NO1C1P0 |  | No.13 | male | 72 | NO3C0P0 |
| No.14 | female | 89 | NO0C0P0 |  | No.14 | female | 67 | NO3C0P0 |
| No.15 | female | 67 | NO0C0P1 |  | No.15 | male | 83 | NO4C0P0 |
| No.16 | male | 70 | NO0C0P0 |  | No.16 | male | 68 | NO3C0P0 |
| No.17 | female | 69 | NO0C0P0 |  | No.17 | female | 69 | NO2C0P0 |
| No.18 | male | 64 | NO0C1P0 |  | No.18 | female | 68 | NO3C0P0 |
| No.19 | female | 81 | NO0C0P0 |  | No.19 | male | 70 | NO5C0P0 |
| No.20 | female | 76 | NO0C0P0 |  | No.20 | male | 69 | NO3C0P0 |
| No.21 | male | 63 | NO0C1P0 |  | No.21 | female | 78 | NO3C0P0 |
| No.22 | female | 68 | NO0C0P1 |  | No.22 | male | 68 | NO4C0P0 |
| No.23 | female | 50 | NO0C0P0 |  | No.23 | female | 67 | NO4C0P0 |
| No.24 | female | 68 | NO0C1P0 |  | No.24 | female | 68 | NO4C0P0 |
| No.25 | male | 71 | NO0C0P0 |  | No.25 | male | 65 | NO3C0P0 |
| No.26 | male | 59 | NO0C0P0 |  | No.26 | male | 76 | NO3C0P0 |
| No.27 | female | 67 | NO0C0P1 |  | No.27 | male | 67 | NO4C0P0 |
| No.28 | male | 68 | NO0C1P0 |  | No.28 | female | 89 | NO3C0P0 |
| No.29 | male | 69 | NO0C0P0 |  | No.29 | male | 69 | NO2C0P0 |
| No.30 | female | 69 | NO0C0P0 |  | No.30 | female | 69 | NO3C0P0 |

Supplemental Table 1.The grade of lens opacity and identification codes of Controls and ARNCs

.
