# Supplementary figures and images for: Ultraviolet-B induces ERCC6 repression in lens epithelium cells of age-related nuclear cataract through coordinated DNA hypermethylation and histone deacetylation
Source: Clin Epigenetics. 2016 May 26;8:62. doi: 10.1186/s13148-016-0229-y (PMC4880862; doi:10.1186/s13148-016-0229-y)

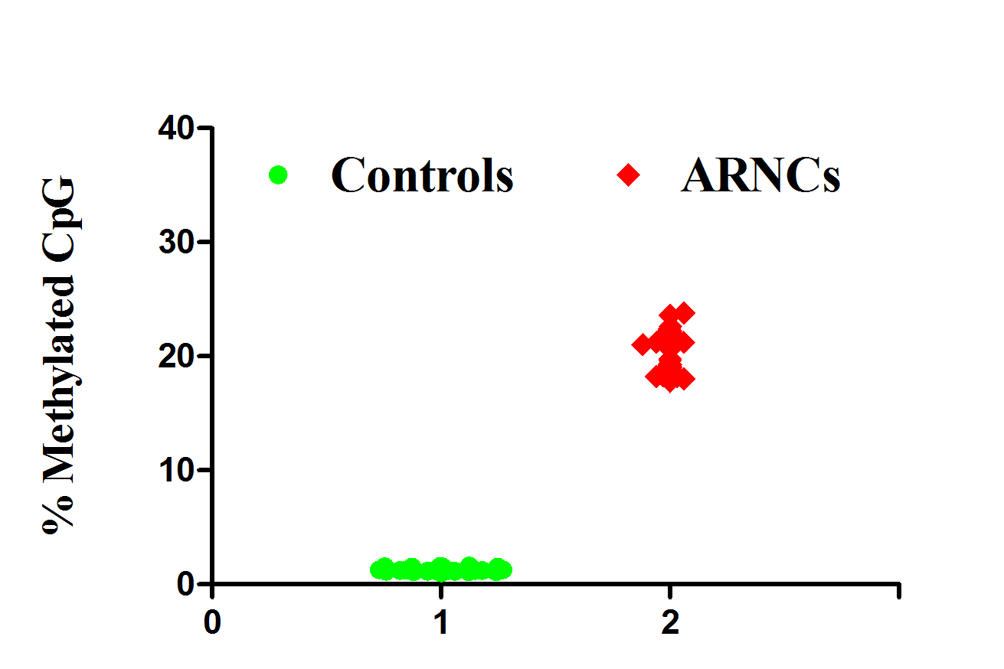

Supplement: Additional file 2: Figure S1. — In LECs of ARNCs, the CpG site 8 displayed hypermethylation compared to the controls (n = 30, respectively) (TIF 1989 kb) [file 13148_2016_229_MOESM2_ESM.tif]
